# The Glial Cells Respond to Spinal Cord Injury

**DOI:** 10.3389/fneur.2022.844497

**Published:** 2022-05-06

**Authors:** Ruideng Wang, Rubing Zhou, Zhengyang Chen, Shan Gao, Fang Zhou

**Affiliations:** Department of Orthopedics, Peking University Third Hospital, Beijing, China

**Keywords:** spinal cord injury, glial cells, reactive astrocytes, microglia, neuroinflammation, remyelination

## Abstract

It is been over 100 years since glial cells were discovered by Virchow. Since then, a great deal of research was carried out to specify these further roles and properties of glial cells in central nervous system (CNS). As it is well-known that glial cells, such as astrocytes, microglia, oligodendrocytes (OLs), and oligodendrocyte progenitor cells (OPCs) play an important role in supporting and enabling the effective nervous system function in CNS. After spinal cord injury (SCI), these glial cells play different roles in SCI and repair. In this review, we will discuss in detail about the role of glial cells in the healthy CNS and how they respond to SCI.

## Introduction

Spinal cord injury (SCI) is a devastating and debilitating neurological and pathological condition with temporary or permanent major motor, sensory and autonomic dysfunctions. It is estimated that there are about 250,000~500,000 people suffering from SCI around the world every year. Besides, ~90% of these cases are caused by traumatic factors, despite the proportion of non-traumatic SCI appears to be growing (1). People with SCI are 2–5 times more likely to die prematurely than people without SCI. Meanwhile, these people with SCI have worse survival rates in low- and middle-income countries. In recent years, more and more studies have begun to reveal the pathophysiology, molecular mechanisms, and possible therapeutic strategies of spinal cord injury. Over the past 50 years, it is gradually realized that glial cells have critical roles in health and disease. Glial cells were first postulated by Virchow in the 19th century and called this unique tissue “Nervenkitt” (2). With time, scientists have been committed to specify these further roles and properties of glial cells in the central nervous system (CNS). The glial cells include four major groups: astrocytes, microglia, oligodendrocytes (OLs), and oligodendrocyte progenitor cells (OPCs). A large number of studies show that these glial cells play an important role in SCI. In this review, we will discuss how these glial cells function in the healthy CNS and respond to SCI.

## Glial Cells Are Vital in Healthy CNS

Glial cells play a vital role in supporting and enabling effective nervous system function in the healthy CNS. During the development of the CNS, glial cells can constitute a cellular framework that contributes to the development of the nervous system, and induce the survival and differentiation of neuron. The main glial cells types include astrocytes, microglial, OLs, and OPCs. They cooperate with each other and perform different important functions in CNS (Table 1).

**Table 1:** Function of glial cells in the healthy CNS.

**Glial cells types**	**Function in CNS**
Astrocytes	Construction of BBB and BSB, regulating blood flow (1)
	Formation, function, and connection of synapses (2)
	Synthesis and maintenance of the ECM (3)
	Neuronal development, migration and differentiation, function (4)
	Energy provision (5)
	Fluid and ion homeostasis (6)
Microglia	Guide neurons and axons in forming prenatal circuits (7)
	Control synaptic density, connectivity and plasticity (8)
	Phagocytose cellular and myelin components (9)
	Regulate development and responses of neuron and other glial cells (10)
OLs	Myelination of Axons and speed conduction velocity (11, 12)
	Support the function and survival of axons (13, 14)
	Information processing (15)
OPCs	Differentiate into oligodendrocytes (12)
	Modulate neuronal activity (16)
	Immunomodulatory capacity (12)

*BBB, blood brain barrier; BSB, blood spinal barrier; ECM, extracellular matrix; OLs, oligodendrocytes; OPCs, oligodendrocyte progenitor cells*.

## Astrocytes in The Healthy CNS

Astrocytes are the most abundant glial cells in CNS that have a large amount of complicated and fundamental functions in the healthy CNS. According to the differences in their cellular morphologies and anatomical locations, astrocytes are divided into two types: protoplasmic astrocytes with the morphological feature of several stem branches are found in gray matter while fibrous astrocytes with the morphological feature of many long fiber-like processes are found in white matter (3). In addition, both astrocyte subtypes have critical roles in health and disease. Astrocytes contribute to the construction of blood-brain barrier (BBB) and blood-spinal barrier (BSB) by combining with endothelial cells and perivascular pericytes through astrocytic endfeet (4–6). Previous studies have indicated that astrocytic endfeet show specialized feature characteristic as the astrocytic endfeet membrane expresses a large number of water channel aquaporin 4 (AQP4) and the Kir4.1 K+ channel, which is important for the properties of BBB (7–9). The Kir4.1 and AQP4 both bind to α-Syntrophin that could contribute to the inductive influence on BBB (10). Astrocytes are proved to produce a series of humoral agents, such as glial cell line-derived neurotrophic factor (GDNF), transforming growth factor-β (TGFβ), and angiopoetin-1 that can induce the aspects of BBB phenotypes (11–13). What's more, it is now recognized that the control of blood flow in brain is mediated by astrocytes. Neuronal activities may result in releasing potassium ions from astrocytic endfeet, extracellular K+ concentration can dilate the vessels through hyperpolarizing smooth muscle cells (14). The rise of Ca^2+^ concentration in astrocytic endfeet can also constrict vessels (15).

Astrocytes contribute to the formation, function, and connection of synapses as astrocytes have a close connection with synapses. The “tripartite synapse” concept was first described by Alfonso Araque, it includes the classic pre- and post-synaptic neuronal structures and astrocytes which should be viewed as integral modulatory elements of tripartite synapses (16). The role of astrocytes in synapses formation was first studied in 1995. Meyer-Franke et al. observed that retinal ganglion cells (RGCs) make very few synapses by purifying and culturing RGC neurons, however, RGCs can make many synapses if they are cultured in an astrocyte feeder layer or a culture medium that is previously conditioned by astrocytes (17). On the basis of the RGC culture system, subsequent studies identified that multiple factors secreted by astrocytes could control the formation of synapses. Thrombospondins (TSPs), the extracellular matrix (ECM) proteins secreted by astrocytes, have been proved to contribute to the formation of synapses (18, 19). By adding purified TSPs to cultured neurons greatly increased the number of synapses. In addition, Cagla Eroglu et al. showed that the von Willebrand factor A (VWF-A) domain of the calcium channel subunit α2δ1 interacts with the EGF-like receptors common to all TSPs which enhanced synaptogenesis both *in vitro* and *in vivo* (20). Hevin, another synaptogenic protein secreted by astrocytes, also induces an increase in the number of structural synapses by bridging presynaptic neurexin-1alpha (NRX1α) (21). Astrocytes can control the specific aspects of synapses function through many different signals, such as positive [cholesterol, glypican 4,6, ECM, tumor necrosis factor a (TNF-a)] and negative (SPARC, TSP) signals (22–27). For example, astrocyte-secreted cholesterol plays an important role in regulating the glutamatergic presynaptic function by complexing to apolipoprotein E-containing lipoproteins (27). Besides, astrocyte-secreted glypican 4/6 has an ability to upregulate the surface level of alpha-amino-3-hydroxy-5-methyl isoxazole propionic acid (AMPA) receptors (AMPARs) at synapses and increase the synaptic activity in neurons (26). As we all know, synapses can undergo rapid formation and elimination under certain conditions. Recent studies have identified some potential mechanisms, such as direct and indirect role of astrocytes in mediating synapses elimination. Microglia have been shown to recognize and phagocytose C1q/C3-coated synapses (28), and astrocytes would express TGF-β to induce the C1q expression which is critical for the phagocytic functions of microglia, and finally astrocytes mediate microglial-dependent synapses elimination (29). Meanwhile, astrocytes contribute to synapses elimination through MEGF10 and MERTK pathways (30).

Astrocytes are actively involved in the synthesis and maintenance of the ECM by secreting various substances in CNS. Tenascin-C, a glycoprotein, is expressed by astrocytes which can regulate cell growth, adhesion, and migration (31). Besides, astrocytes produce a large number of proteoglycans, such as chondroitin sulfate proteoglycans (CSPGs), which are suited for regulating neural development (32).

Other aspects of the role in CNS, such as astrocytes can store glycogen granules and make important contributions to the metabolism in CNS (33). The astrocyte-neuron lactate shuttle hypothesis which explains how astrocytes support neurons energy metabolism in detail. Glutamate released by neurons during the neuronal activity can bind to glutamate transporters (GLT-1), expressed by astrocytes, which mediate astrocytes taking up glucose from the blood circulation *via* glucose transporters (GLUT1). Then, glucose is subsequently metabolized to lactate and pyruvate. On the one hand, intracellular lactate can be shuttled to extracellular matrix *via* monocarboxylate transporter (MCT) 1 and MCT4 expressed by astrocytes and then could be absorbed by neurons through neuronal MCT2. Neuronal lactate can participate in the neuronal cell energy metabolism and promote ATP synthesis in the mitochondria directly or after conversion to pyruvate (34, 35). Similarly, ammonium (${\text{NH}}_{4}^{+}$) released by neurons increase lactate levels in astrocytes which can be shuttled to neurons (36). In summary, as astrocytes possess unique cellular properties, they play a vital role in the function and integrity of CNS (Table 1).

## Microglia Functions in The Healthy CNS

Debate on microglial origin still continues in this field, recent studies showed that microglia were derived from erythromyeloid precursors in the yolk sac through Pu.1- and Irf8-dependent pathways (37). Microglia are crucial for the development of CNS. They arise around the same time as neurons and critically contribute to the establishment of complex neuronal networks. During the early development of CNS, microglia act as guidepost cells to guide neurons and axons to form prenatal circuits (38). Moreover, microglia are involved in the regulation of surrounding cellular milieu by secreting trophic factors [brain-derived neurotrophic factor (BDNF) (39), insulin-like growth factor-1 (IGF-1) (40), and hepatocyte growth factor (HGF) (41)] which could promote the survival of neurons. For instance, the best-known trophic factors, IGF-1, can enhance the survival of cortical neurons. On contrary, inhibiting IGF-1 signaling (minocycline, CD11b-DTR, and Cx3cr1^GFP/GFP^) would result in the cell death in layer V (40). Besides, microglia are the sensors of damage as they can phagocytose apoptotic neuron driven by both TAM receptor ligands Gas6 and protein S (42). Additionally, they engulf excess new born neural progenitor cells *via* primary phagocytosis which is beneficial to the homeostasis during the development of CNS (43).

Microglia play an important role in the control of synaptic density, connectivity, and plasticity. Microglia can selectively remove synapses from injured neurons which is termed “synaptic stripping” (44, 45). This process is identified to be mediated through several mechanisms. C3 receptors (CR3) expressed by microglia can bind to C1q and C3, the complement proteins expressed by damaged cells, which could lead the microglia to be involved in the active removal or “stripping” of these synaptic contacts and finally contribute to synaptic elimination (46). Microglia can also activate “synaptic stripping” through the fractalkine/CX3CR1 signaling pathway (47). Except for the receptor binding mode, microglia can also shape the strength and plasticity of synapses by releasing reactive oxygen species (ROS) (48), nitric oxide (NO) (49), TNF-α (50) as well as neurotrophic factors [BDNF (51)]. For example, microglia-derived BDNF activates Trk in spinal neurons that could impact synapse activity (52). Above all, microglia are vital for neuronal health and survival during the development of CNS (Table 1).

## OLs and OPCs Functions in The Healthy CNS

Another major glial cell type is OLs, generated from OPCs, are fundamental to the myelin formation in CNS. The newborn OPCs can express DM-20 during embryonic development, and first appear in a restricted region of the embryonic ventral neural tube at embryonic day 12.5 in mice (53). Then, they finally differentiate into OLs through a complicated process. Importantly, OPCs are observed to differentiate into OLs throughout development and adulthood. Except for differentiating into OLs, OPCs can tile throughout the entire CNS and constitute ~5% of all cells (54). The fate of OPCs to keep as precursor cells or differentiate into OLs is influenced by many factors, such as mechanical environment and extracellular matrix elasticity (55–57). OPCs continue to be precursor cells by self-renewal to achieve homeostasis in CNS. Besides, OPCs express GABA receptors, kainite glutamate receptors, and AMPA receptors to form neuron-OPC synapses which modulate the neuronal activity (58, 59).

Oligodendrocytes are crucial for maintaining the function and integrity of axons. The most important function of OLs is to generate myelin sheath, as we all know that myelin sheath is an extension structure of the OLs plasma membrane wrapping the nerve axons. Myelination is a complex and tightly regulated process: OLs in the growth zone of CNS undergo proliferation under certain factors, then contact and arrange along the axon, respectively. The inner and outer plasma membrane wrapping the nerve axons interact with each other through cytoplasmic channels which pushes the inner plasma membrane layer after layer to generate the compact myelin. Once the appropriate number of plasma membrane wrapping per axon is generated, this process is called myelination (60). Functionally, the myelin sheath enables fast and efficient nerve conduction in the nervous system and provides metabolic support to the axons (61).

Oligodendrocytes have a physiological role in supporting the function and survival of axons that is independent of myelination. In the absence of PLP and DM20, the membrane proteolipids of myelin sheath that are integral for myelinated axons, myelination is not disrupted but with subsequently widespread axonal dysfunction (62). Subsequent studies found that PLP/DM20 was important for OLs in supporting the axonal energy metabolism (63, 64). With the further study, it is now well-recognized that OLs are essential for supporting the axons energy metabolism (65). The mechanisms how OLs provide neuronal metabolic support are described in detail as following. OLs can express a large number of MCT1, which can mediate metabolic support to neurons by co-transporting lactate and pyruvate (66). OLs can take up glucose from the extracellular matrix *via* GLUT1 expressed by OLs and then convert glucose into lactate and pyruvate by glycolysis. Besides, glutamate released by neuron after neuronal activity can bind to NMDA receptors (NMDARs) expressed by OLs which subsequently result in an increased glucose uptake as well as more lactate and pyruvate production in OLs (67). Moreover, the gap junctions between astrocytes and OLs may contribute to OLs metabolic support as lactate and glucose derived from astrocytes could be shuttled into OLs through gap junctions, such as Cx32-Cx30, Cx32-Cx26, Cx47-Cx30, and Cx47-Cx43 (68–70). All the functions of OLs and OPCs in healthy CNS are shown in Table 1.

## Glial Cells Respond To SCI

As discussed above, glial cells, such as astrocytes, microglia, OLs, and OPCs all are crucial for the development of CNS and maintaining homeostasis in healthy CNS. They have different and vital physiological functions for the CNS due to their cytological properties and cellular interactions. After SCI, the noxious mechanical forces cause tissue damage, such as cells death and disrupt the homeostasis of local CNS, as a result, these events trigger diverse multi-cellular responses and can lead either to the neural repair or secondary cellular injury. Glial cells exhibit various pathophysiological functions to repair the damage and maintain local microenvironment homeostasis due to various internal and external factors after SCI. Next, we will describe in detail the response of various glial cells to SCI.

## Astrocytes: Reactive Astrocytes and Glial Scar Formation

Astrocytes, as discussed above, are essential to maintain the homeostasis in healthy CNS. Similarly, astrocytes also play an important role after SCI. After SCI, various intrinsic and extrinsic factors subsequently regulate astrocytes into reactive astrocytes with significant morphological, phenotypical, and functional changes, such changes are mainly based on different factors, such as the injury severity, the injury time, and the distance of astrocytes to the lesion. Reactive astrocytes have characteristics in morphology, such as cellular hypertrophy, thicker processes, and increased expression of intermediate filament proteins. Besides, the degree of changes are proportional to the stimulus intensity (71). On the basis of discrete gene-expression identifiers and functions, different types of reactive astrocytes have been recognized, such as A1, A2, and scar-forming astrocytes (72, 73). For example, complement component 3 is highly expressed by A1 astrocytes, and S100A10 is a specific hallmark for A2 astrocytes while type I collagen for scar-forming astrocytes (73, 74). Compared with the normal astrocytes, accumulating evidence suggests that reactive astrocytes show various abnormal functions, such as releasing proinflammatory chemokines and cytokines (71).

### Molecules and Signaling Pathways Implicated in Formation of Reactive Astrocytes

Mechanical forces usually cause direct damage to the normal tissue and disrupt local homeostasis when patients or animals undergo SCI, which on the other hand triggers multitudinous multi-cellular responses. Although it is incompletely understood how mechanical forces and damaged tissues initially trigger the activation of astrocytes after SCI. The previous study has identified that astrocytes are susceptible to membrane distortions and debris (75). Traumatic membrane deformation could activate mechanosensitive ion channels and result in the rapid influx of extracellular calcium and sodium in astrocytes (76–78). Other studies show that plasma membrane stretching can rise the release of intracellular calcium and ATP *via* extracellular signal-regulated protein kinase (ERK) and PKB/Akt signaling pathways (79, 80). Besides, astrocytes may also release endothelin-1 (ET-1), isoprostanes, and matrix metalloproteinases 9 (MMP-9) after stretch-induced injury (81, 82). More studies are needed to have a deeper understanding of these.

Accumulating studies have identified that a lot of molecules, such as chemokines, cytokines, transcription factors, and growth factors are the mediators for the activation of astrocytes (factors are shown in Table 2). For example, proinflammatory cytokines, such as TNF-α, interleukin (IL)-6, and IL-1β initially trigger the reactivity of astrocytes during the acute phase after SCI while other molecules maintain astrocytes reactivity in the later stages (83–85). Additionally, it is worth mentioning that reactive astrocytes can release triggering molecules, such as TNF-α, IL-6, and MMP-9, which in turn activate more astrocytes (86). Besides, other glial cells, such as activated microglia, are identified to induce the activation of astrocytes by secreting various factors, such as Il-1α, TNF, and C1q (87). Other related molecules involved in the activation of astrocytes are shown in Table 2.

**Table 2:** The activation of astrocytes.

**Factors**	**Signaling pathways**	**Molecules and gene expression**
Membrane stretching (17–19) ATP (19), debris (20) IL-1β, IL-1α, IL-2, IL-6, IL-10, IL-17, TNF-α, IFN-γ, CNTF, TGFβ1, INFγ, IL-2, LIF, C1q, oncostatin M, SHH (21–26) Glutamate, norepinephrine (27) NO, ROS (26) MCP-1, FGF-2, IGF, MMP-9, Sox9 (4) Amyloid-beta (28), α-synuclein (29) Estrogens (30), glucocorticoids (31) LPS, Toll-like receptor ligands (32) Laminin, fibronectin (33) Erythropoietin, ET-1 (34)	STAT3 signaling (35) NFκB signaling (36) TGF-β signaling (37) JNK/c-Jun signaling (34) MAPK Signaling (38) Olig2 (39) SOC3 (40) RhoA (4) Smads (4) cAMP (41) IGF1-calcineurin (42)	CCL2, CCL3, CCL4, CCL5, CXCL1, CXCL2, CXCL10, CCL12, CXCL20 (37, 43, 44) VEGF, FGF-2, BDNF, GDNF (24, 45, 46) IL-1β, IL-6, IL-10, TNF-α, INF-γ, TGF-α, TGF-β, CNTF, LIF, CLCF1 (23, 37, 43, 47, 48) CSPGs, IGFBP6, BMP, connective tissue growth factor, collagen I, fibronectin, MMP-9 (49–52) ROS, NO, NOS (53–55) GABA, glutamate, d-serine (56–58) Nestin, vimentin, GFAP (49, 52) EGFR, KCa3.1, AQP4 (59–61) STAT3, NF-κB, Olig2, SOX9, mTOR, SOCS-1, SOCS-3 (40, 47, 48) Adenosine, glutathione (4)

*IL, interleukin; CNTF, ciliary neurotrophic factor; LIF, leukemia inhibitory factor; SHH, Sonic hedgehog; MCP-1, Monocyte chemoattractant protein-1; FGF-2, fibroblast growth factor-2; NO, nitric oxide; ROS, reactive oxygen species; IGF, insulin-like growth factor; LPS, lipopolysaccharide; MMP-9, matrixmetalloproteinase-9; ET-1, endothelin-1; VEGF, vascular endothelial growth factor; BDNF, brain derived neurotrophic factor; GDNF, glial cell derived neurotrophic factor; CSPGs, chondroitin sulfate proteoglycans; BMF, bone morphogenetic protein; GFAP, glial fibrillary acidic protein; EGFR, epidermal growth factor receptor*.

Many signaling pathways are closely involved in the activation of astrocytes, such as STAT3, TGF-β, NF-κB, JNK/c-Jun, and MAPK (more signaling ways are shown in Table 2). Here we will mainly introduce TGF-β and STAT3 signaling pathways. The STAT3 signaling pathway is one of the most important signaling pathways to mediate the formation of reactive astrocytes. Mice with STAT3 knock-out in astrocytes showed the attenuated upregulation of GFAP, unsuccessful cell hypertrophy, and failed scar formation after SCI (88). Other groups also identified that selective STAT3 deletion in mice could limit the migration of astrocytes and result in the widespread infiltration of inflammatory cells, degeneration of neurons, and demyelination of axons that can lead to severe motor deficits. However, by the activation of STAT3 signaling pathway, they observed that reactive astrocytes migrated rapidly around the lesion and secluded inflammatory cells that lead to a notable improvement in functional recovery (89). These results provided a potential intervention target of STAT3 signaling pathway in the treatment of SCI. TGF-β signaling pathway greatly contributes to the formation of reactive astrocytes. As discussed above, TGF-β is a key upstream trigger in the formation of reactive astrocytes. The previous study has shown that TGF-β could increase the expression of anti-regenerative molecules, such as CSPGs, laminin, and fibronectin by several-fold in reactive astrocytes (90). Interestingly, fibrinogen could act as a stimulating factor which can activate TGF-β signaling pathway, as a result, it could induce the activation of astrocytes and formation of CSPGs (91). In addition, it could induce astrogliosis by injecting fibrinogen into the mouse cortex (91). On contrary, with the genetical ablation of fibrinogen in mice, they found inhibited TGF-β activation and hampered glial scar formation (91). Other signaling pathways are shown in Table 2.

### Reactive Astrocytes Expression Change and Their Functions

Recent years, numerous studies have identified that the activation of astrocytes could lead to the change of functions with releasing a range of molecules, such as cytokines [TNF-α, IL-6, IL-10, IL-1β, etc. (85, 92, 93)], chemokines [CCL2, CCL3, etc. (94, 95)], growth factors [BDNF, GDNF, etc. (96, 97)], toxic amino acids [GABA and glutamate (98, 99)], extracellular matrix [CSPGs, collagen I, fibronectin, MMP-9, etc. (100–102)], and intermediate filaments [Nestin, vimentin, and GFAP (100, 102)], which would have a significant influence on the spinal cord microenvironment after SCI (Table 2). The molecules released by reactive astrocytes can activate more normal astrocytes into reactive astrocytes and contribute to glial scar forming. On the other hand, they also affect other cells, such as neurons, OPCs, and microglia through a variety of complexed effects (71).

Over the past years, reactive astrocytes were thought to be detrimental for recovery after SCI. However, recent studies have identified that reactive astrocytes also contribute to SCI repair. Here, we will discuss the beneficial and detrimental effects of reactive astrocytes after SCI (Table 3).

**Table 3:** Positive and negative influence of reactive astrogliosis.

**Positive influence of reactive astrocytes**
Seclude inflammatory cells and limit the extent of inflammation (62)
Repair damaged BSB and modulate blood flow (62)
Clearance of debris, alleviation of glutamate excitotoxicity (53, 63)
Mediate neuroimmune response (32)
Formation of glial scar (64, 65)
Defend against oxidative stress (64)
Contribute to remyelination (66)
**Negative influence of reactive astrocytes**
Obstruct axon growth, facilitate axon degeneration (67)
Formation of glial scar (64)
Inhibition in NPCs and OPCs (68)
Contributes to the development and persistence of chronic pain (69)

Reactive astrocytes are considered to be a defense mechanism of astrocytes responding to SCI. After SCI, BBB breaks down and becomes leaky to endogenous and exogenous blood-borne macromolecules that can result in disastrous consequence. These changes will mediate reactive astrocytes to upregulate Sonic hedgehog (SHH) and activate signaling cascades to repair the tight junctions of the BBB (103). Interestingly, with the absence of reactive astrocytes, it was failure in repairing the damaged BBB (104). At acute stage after SCI, reactive astrocytes migrate rapidly around the lesion to seclude inflammatory cells and limit the extent of inflammation that has a notable improvement in functional recovery (89). Further, Jill et al. found significantly increased and prolonged infiltration of inflammatory cells around the lesion with selective and conditional reactive astrocytes ablation in mice (104). Various endogenous and exogenous factors result in the release and accumulation of cell debris and neurotoxic factors in the extracellular spaces after SCI. Recently, reactive astrocytes were identified to play a crucial role in removing these cell debris and neurotoxic factors. More importantly, reactive astrocytes have the ability to phagocytose dead cells *in vitro* and *in vivo via* the upregulation of ABCA1 (105, 106). Reactive astrocytes can also reduce the impact of glutamate excitotoxicity on neurons and OPCs by clearing excess glutamate from the blood or necrotic neuronal cell death (107). Besides, reactive astrocytes can affect immune cells through releasing various molecules, such as TNF-α, TGF-β, and proteoglycans. CSPGs have a close relationship with immune activity as they can recruit chemokines and growth factors that enhances the connection of immune cells (71).

Glial scar formation has been recognized for many years. After SCI, inflammatory cells (macrophages, neutrophils, and lymphocytes), fibrotic cells, and other cells, such as pericytes, fibroblasts, and OPCs migrate rapidly into the lesion, and subsequently newly proliferated, elongated reactive astrocytes come around the lesion to form a border which could separate necrotic tissue from healthy tissue (108–110). The border formed by reactive astrocytes can limit further expansion of the lesion and restrict inflammatory cells within damaged tissue that will protect the surrounding viable neural tissue from secondary damage (111). Further, selective inhibition of astrocyte reactivity results in the widespread propagation of inflammatory cells beyond the lesion.

In addition to the above protective effects, reactive astrocytes also have detrimental effects. As discussed above, reactive astrocytes can form a physical barrier to confine the lesion, however, it can also obstruct axonal growth. Besides, reactive astrocytes secrete inhibitory proteins, such as CSPGs, which are considered to be the major inhibitors of axonal regeneration. CSPGs derived from reactive astrocytes inhibit the growth of axons *in vitro*, and axonal regeneration is observed to stop at CSPG-rich regions *in vivo*. On the contrary, Chondroitinase ABC, by removing CSPG glycosaminoglycan (GAG) chains, attenuates the inhibitory activity of CSPGs, which is shown to facilitate axonal regeneration and functional recovery (112). Further, Hyunjung Lee et al. found that using thermostabilized Chondroitinase ABC through a hydrogel-microtube scaffold system could enhance the axonal regrowth, sprouting, and improve functional recovery after SCI (113). Additionally, other studies have shown that CSPGs inhibited axonal regeneration while the inhibition of CSPGs could improve functional recovery (114).

Reactive astrocytes play a modulatory role in NPCs and OPCs post-SCI. OPCs have extremely powerful ability in remyelination as they can proliferate and differentiate into OLs that will replenish a large number of lost OLs after SCI. Recently, Justin R Siebert et al. have discovered that astrocytes-derived CSPGs highly inhibited the migration and differentiation of OPCs *in vitro*, and the number of OPCs surrounding the lesion significantly increased when treated with the enzyme chondroitinase ABC (115). Other study also proved that CSPGs had a dampening effect on the outgrowth and differentiation of OPCs, and treated with chondroitinase ABC could completely eliminate this inhibition (116). In addition to CSPGs, other molecules, such as BMP and ET-1 released by reactive astrocytes can also inhibit the differentiation of OPCs and finally influence remyelination (117, 118). Besides, reactive astrocytes have a role in inhibiting the neuronal differentiation of NPCs by expressing insulin-like growth factor binding protein 6 (IGFBP6) and CSPGs (119).

## Microglia/Macrophages: Neuroinflammation

Microglia/macrophages maybe the most potent modulators to launch the innate immune response after SCI. As discussed above, we know that microglia are resident in CNS while macrophages derive from the periphery. However, activated microglia and macrophages are difficult to distinguish through the morphology or antigenic markers following CNS injury, so they are referred as microglia/macrophages. Over the past years, the studies have revealed that microglia/macrophages had both the detrimental and beneficial effects on neurological recovery due to their different phenotypes at different stages after SCI (120).

### Microglia/Macrophages Phenotypes

Microglia/macrophages phenotypes are mainly determined by the focal lesion and new stimuli can change the phenotypes. It is now well-acknowledged that microglia/macrophages are activated into different functional phenotypes after SCI. M1/M2 dichotomy is the earliest and simplest concept. M1 macrophages (or ‘classically' activated macrophages) are activated by the prototypical T helper 1 cytokine (TH1), interferon-γ (IFNγ), and lipopolysaccharide (LPS), which typically release inflammatory cytokines (IL-1, IL-6, TNFα, etc.), chemokines (CCL8, CCL 15, CXCL 10, CXCL 11, etc.), and the high levels of oxidative metabolites (ROS and NOS). On the contrast, M2 macrophages (or “alternatively” activated macrophages) are activated by the prototypic TH2 cytokine IL-4 and IL-13, which can produce numerous protective factors (TGFβ, IL-10, IL-1Ra, etc.) and clear cellular debris (120–122). However, the status and functional phenotypes of microglia/macrophages are much more complicated *in vivo*. Accumulating studies have identified the multiformity in M2 phenotype subpopulations, such as M2a, M2b, and M2c phenotypes, each phenotype is characterized by unique physiological features and distinct biological functions (121). Nowadays, microglia/macrophages in many other situations did not show a clear M1 or M2 phenotype or showed phenotypic plasticity during the disease progression. Single cell techniques and other new tools are, contributing to the understanding of polarization heterogeneity (123). By single-cell analysis, Lindsay M Milich at el. identified four microglial subtypes in the injured mouse spinal cord, which were labeled homeostatic, inflammatory, dividing, and migrating microglia. Homeostatic microglia were identified by several annotated markers of steady-state microglia, such as P2ry12, Siglech, and Tmem119. Inflammatory microglia were identified by the low expression of purinergic receptor P2ry12 and increased expression of Igf1. Dividing microglia expressed low levels of P2ry12, increased expression of Msr1, and high levels of cell cycle–related genes, such as Cdk1. Migrating microglia had the low levels of P2ry12, and the high levels of Msr1 and the growth factor Igf1 (124). Besides, two macrophage subtypes were named chemotaxis-inducing macrophages and inflammatory macrophages in addition to the border-associated macrophages based on their gene ontology terms. Both subtypes expressed the lysosomal gene Cd63, however, chemotaxis-inducing macrophages preferentially express heme oxygenase Hmox1 while inflammatory macrophages express *Apoe* (124, 125).

### Microglia/Macrophages Respond to SCI

Activated microglia could release a large number of pro-inflammatory cytokines, chemokines, and other cytotoxic factors after SCI. They respond to SCI within minutes by producing pro-inflammatory molecules which can lead to the influx of multiple inflammatory cells from the circulation. Neutrophils are the first circulating leukocytes to infiltrate into the lesion and are prominently located in severely damaged site (126, 127). Besides, peripheral macrophages will infiltrate into the lesion and help clear apoptotic cells (127). However, these neutrophils and macrophages may be destructive to the lesion as they can produce various molecules, such as MMP-9 and disrupt the functions of the BSB (128). Besides, T and B lymphocytes are found to infiltrate into the injured lesion and cause a systemic autoimmune response (129). Here, we will mainly discuss the harmful and beneficial effects of neuroinflammation induced by activated microglia/macrophages after SCI.

Activated M1 microglia/macrophages induce neurons death and contribute to the secondary damage by releasing pro-inflammatory factors, such as IL-1β, IL-6, TNF-α, CCL5, and iNOS. Here we mainly elaborate IL-1β and TNF-α that play a detrimental role after SCI. IL-1β expressed by astrocytes and microglia was detected to reach peak at 12 h after SCI in rodents (130). IL-1β and TNF-α were proved to involve in the recruitment and activation of peripheral immune cells and the activation of astrocytes and microglia. In rats, the infusion of IL-1β markedly enhanced the cortical neuronal loss, on the contrast, it could significantly inhibit neuronal damage by IL-1 receptor antagonist (IL-1ra) (131). Other study also identified that IL-1β contributed to ischemic brain damage while IL-1ra markedly protected the focal cerebral from ischemia in the rat (132). TNF-α, another proinflammatory cytokine, expressed mainly by activated microglia/macrophages, contributes to neuronal cells death after SCI by binding to TNFRI and TNFRII (133). In addition, soluble TNFRI, which can compete with TNF-α by binding to TNFR, eventually reduces the neuronal cells death (133). Tiziana Genovese et al. indicated that the genetic inhibition of TNF-α significantly reduced the degree of inflammation, tissue injury, and apoptosis in an experimental model of spinal cord trauma (134). Besides, overexpressing TNF-α was shown to mediate OLs, OPCs death, and myelin vacuolization which could finally result in spontaneous demyelination (135).

Activated M2 microglia/macrophages have anti-inflammatory and neuroprotective effects by increasing the expression of anti-inflammatory molecules, such as IL-10, TGF-β, IGF-1, and BNDF. For example, IL-10 shows a wide range of regulatory activities in response to SCI. Tiziana Genovese et al. found that there was a significant augmentation of TNF-α, IL-1β and S100β which worsened the recovery of limb function in IL-10 KO mice (136). Recently, the group of Jessica Y Chen delivered IL-10 into mice SCI model by loading an implantable biomaterial scaffold. They observed that IL-10 could significantly reduce damage to tissue and improve subsequent motor deficits (137). IGF-I is a potent neurotrophic factor released by activated microglia/macrophages with anti-inflammatory response. The previous study showed that IGF-I gene transfer after SCI could inhibit the loss of neurons and significantly improve the neurological dysfunction (138). Besides, other study showed that BDNF and IGF-I could significantly enhance neuroprotective effects, such as repairing BSCB damage, alleviating edema, and cells injury by the downregulation of nNOS after SCI in rat model (139).

## OLs and OPCs: Demyelination and Remyelination

### OLs and Demyelination

In addition to the immediate trauma damage, there is a prolonged secondary damage after SCI. OLs are quite susceptible to changes in the surrounding microenvironment after SCI which can result in the necrosis, apoptosis, and autophagy of OLs (140–142). Acute OLs death has previously been investigated to occur within 15 min after injury and the number of OLs steadily declined by 7 days post-injury (143, 144). Previous studies have identified several aspects of subsequent damage that can lead to the death of OLs. Ischemia is an apparent reason to result in OLs death in the damaged areas of white matter (145). Ischemia and reperfusion contribute to the formation of free radical, such as reactive oxygen and nitrogen species, and OLs are particularly vulnerable to the oxidative stress. After SCI, ROS (hydroxyl radicals and superoxide) and RNS (nitric oxide, peroxynitrite, and nitrated protein) were detected to be at the increased levels (146–148). By oxidizing protein, lipids and nuclear material, ROS and NOS damage OLs which results in the necrosis and apoptosis of OLs. Besides, excitotoxicity is another major factor leading to the OLs death after SCI. The glutamate will reach a toxic level after SCI that can lead to the OLs death *in vitro* and *in vivo*. Glutamate binding to AMPA/kainate glutamate receptors expressed in OLs leads to OLs death *via* receptor overactivation and the specific inhibitors of AMPA receptors can block OLs death (149). As discussed above, extracellular ATP released by multiple cell types after SCI can also contribute to OLs death. ATP is proved to cause OLs death *via* an activation of calcium-permeable P2X(7) and treatment with P2X(7) antagonists reduces demyelination and improve neurological symptoms (150). In addition, recent studies reveal that proinflammatory cytokines potentially contribute to OLs cell death. An overexpression of TNF-α was observed to induce OLs apoptosis which could contribute to the degenerative change and demyelination *via* TNFR1 and TNFR2 expressed in OLs (151). Other cytokines, such as IL-2, IL-1, IFNγ, and proNGF, all contribute to OLs apoptosis (142). In addition to apoptosis and necrosis, autophagy is activated in SCI, and Beclin1, a promoter of autophagy, is highly expressed in OLs (152).

Oligodendrocytes are fundamental to myelin formation as described earlier. The injury or death of OLs results in the degeneration of myelin sheaths and the support of axons by OLs would be disrupted after SCI which eventually lead to the widespread demyelination of spared axons. As a matter of fact, accumulating studies have demonstrated that demyelination indeed occurred in animal models and human after SCI (153, 154). For example, demyelinated axons were seen within 2 weeks after injury in paraplegic domestic animals in previous study (153). More interestingly, the extent of demyelination mainly contingents on the type and severity of injury. The normal myelinated axons are characterized by the regular distribution of sodium and potassium channels, after demyelination, the distribution of sodium and potassium channels is disrupted that contributes to an axonal conduction block (155). Besides, demyelination is identified to increase voltage-gated Na+ channels, which may result in Na^+^ influx during action potential propagation. To eliminate the excess Na^+^, more ATP is required which can disrupt the axonal internal energy balance. Additionally, the excess Na^+^ may lead to axonal Ca^2+^ overload *via* the Na^+^/Ca2^+^ exchangers. These events eventually result in axonal degeneration (156, 157). Besides, the demyelinated axons are vulnerable to damage in the microenvironment after SCI and ultimately lead to axonal degeneration (158).

### OPCs and Remyelination

After SCI, OPCs are multipotential stem cells which can differentiate into remyelinated cells to involve in axonal remyelination and contribute to the glial scar formation. McTigue et al. assessed the proliferation of NG^2+^ cells and OLs by bromodeoxyuridine incorporation and they found increased proliferation of NG^2+^ cells persisting throughout the first 4 weeks post-injury while the number of OLs continuously reduced by 7 days post-injury. However, they detected an increased number of OLs at 14 days post-injury. These results showed that proliferated NG^2+^ cells may differentiate into OLs after injury (159). Besides, the study using fate mapping confirmed that 30% of new OLs responsible for myelin regeneration were derived from OPCs while OPCs differentiate into the majority of myelinating Schwann cells (160). Other group also revealed that OPCs from the PDGFRα-expressing lineage could be transformed into functional myelinating Schwann cells after SCI (161). Moreover, by using genetic fate mapping, Hackett et al. found that ~25% of astrocytes were derived from NG^2+^ cells in the glial scar by 4 weeks after SCI (162). It is worth mentioning that the functions of OPCs are intricately modulated by a complex network including various factors (Table 4). We will not go into further discussion here. In addition to OPCs, the endogenous NPCs can also contribute to OLs replacement as they will be activated and migrate into the lesion after SCI (163, 164).

**Table 4:** Factors regulate remyelination via different effects on OPCs.

**Classifications**	**Factors**	**Effect on OPCs**
Growth factors	PDGF-A; EGF; FGF-2; IGF; Nrg-1 (70–72)	Survival ↑
Neurotrophins	BDNF; NT-3 (73, 74)	Proliferation↑
Chemokines	CXCL1; CXCL12 (74)	Migration ↑
Cytokines	CNTF; LIF; IFN-γ; IL-17A; IL-1β (66, 75, 76)	Differentiation ↑
Transcription	OLIG1; OLIG2 (77)	
factors	SOX5; SOX6; SOX8; SOX9; SOX10 (78, 79)	
	ZFP191; ZFP488 (80)	
	MYT1; MASH1; NKX family; YY1 (81–83)	

*PDGF-A, platelet-derived growth factor; Nrg-1, neuregulin-1; IL, interleukin; BDNF, brain derived neurotrophic factor; FGF-2, fibroblast growth factor-2; IGF, insulin-like growth factor; EGF, epidermal growth factor; NT-3, neurotrophin-3; LIF, leukemia inhibitory factor*.

Remyelination occurs spontaneously on residual axons after SCI. Remyelination is difficult to detect until genetic fate mapping approaches are applied, the scientists can distinguish new myelin from preexisted myelin *via* labeling new myelination. Assinck et al. found that spontaneous remyelination was induced by OLs and myelinating Schwann cells in mice after SCI (160). Besides, other group detected remarkably clear visualization of spontaneously regenerated myelin *in vivo* (165). However, endogenous remyelination was limited due to multi-factors (166). Nashmi et al. found that the spontaneous remyelination in the injured white matter was non-optimal and incomplete because the newly formed myelin around the injured axons was thinner than normal myelinated axons (167). Recent studies have uncovered that multiple factors affected remyelination, such as (1) the myelinating OLs derived from OPCs are inadequate (168), (2) OLs maturation and myelination are limited (142), (3) axonal ensheathment and remyelination is influenced (169), and (4) OPCs, neural progenitor cells (NPCs) are affected by the unfriendly microenvironment (166). Therefore, more endogenous mechanisms of remyelination are needed to be explored.

## Conclusions

Glial cells play a crucial role in maintaining the function and homeostasis of the CNS. Once the homeostasis of the CNS is disrupted, glial cells will respond to the different kinds of damage by multiplying, differentiating, activating, and so on. Nowadays, based on the animal models of SCI, we have gained a better understanding of the pathophysiological changes of glial cells after SCI. For example, after SCI, various factors lead to the activation of astrocytes, which can secrete various molecules, such as cytokines and chemokines in response to SCI. Besides, multicellular and multi-molecular components are involved in forming glial scar that has beneficial and detrimental effects in axonal regeneration and neuro-inflammation. Therefore, an in-depth exploration of the role of glial cells in SCI is conducive to the development of SCI repair strategies. Further studies should develop novel targets and strategies that contribute to the post-SCI reparative responses of glial cells.

## Author Contributions

RW and RZ contributed to the writing of the manuscript. ZC and SG contributed to a systematic literature search. All authors discussed the results and contributed to the final manuscript.

## Funding

This work was supported by the National Natural Science Foundation of China (No. 81971160).

## Conflict of Interest

The authors declare that the research was conducted in the absence of any commercial or financial relationships that could be construed as a potential conflict of interest.

## Publisher's Note

All claims expressed in this article are solely those of the authors and do not necessarily represent those of their affiliated organizations, or those of the publisher, the editors and the reviewers. Any product that may be evaluated in this article, or claim that may be made by its manufacturer, is not guaranteed or endorsed by the publisher.
